# Paraneoplastic Cutaneous Manifestations of Hepatocellular Carcinoma. A Systematic Review and Meta-analysis

**DOI:** 10.7150/jca.88931

**Published:** 2024-01-01

**Authors:** Laith Al-Showbaki, Ahmad A. Toubasi, Dunia Z. Jaber, Mohammad Al Shdifat, Noor Al-Maani, Omar Qudah, Feras Fararjeh, Eitan Amir

**Affiliations:** 1 Division of Hematology and Medical Oncology, Department of Medicine, Jordan University Hospital and School of Medicine, the University of Jordan, Amman, Jordan.; 2 School of Medicine, The University of Jordan, Amman, Jordan.; 3 Division of Dermatology, Department of Medicine, Jordan university Hospital and school of Medicine, The University of Jordan, Amman, Jordan.; 4 Division of Hepatology and gastroenterology, Department of Medicine, Jordan university Hospital and school of Medicine, The University of Jordan, Amman, Jordan.; 5 Division of Medical Oncology and Hematology, Princess Margaret Cancer Centre, University Health Network, University of Toronto, Toronto, ON. Canada.

**Keywords:** Paraneoplastic syndromes, Cutaneous manifestations, Hepatocellular carcinoma, Liver cancer

## Abstract

**Background:** There remains a scarcity of published data on the clinical significance of paraneoplastic cutaneous manifestations in hepatocellular carcinoma (HCC).

**Method:** A systematic search of MEDLINE was performed in December 2022. Inclusion criteria comprised studies reporting on patients with HCC, who had paraneoplastic cutaneous manifestations. Outcomes of interests comprise survival and response to cancer-directed and/or skin directed therapy.

**Results:** A total of 48 studies comprising 60 HCC patients were included in the analysis. The most frequent reported skin abnormalities were dermatomyositis, pityriasis rotunda, and porphyria. Most patients presented with dermatomyositis had underlying viral hepatitis, while all reported porphyria and acanthosis cases were associated with metabolic causes of HCC, such as steatosis. Paraneoplastic skin changes were more common in patients with metastatic disease. Pityriasis Rotunda was associated with the lowest risk of death, (OR: 0.05, 95% CI: 0.003 to 0.89; p = 0.04), while dermatomyositis had a statistically significant higher risk of death (OR: 3.37, 95% CI: 1.01-12.1; p = 0.03). Most patients showed an improvement in their cutaneous abnormalities, following cancer-directed therapy.

**Conclusion:** Paraneoplastic cutaneous manifestations are reported more frequently in patients with a higher burden of disease, especially presence of metastases. Certain cutaneous manifestations have prognostic implication.

## Background

Hepatocellular carcinoma (HCC) is a major cause of cancer related mortality worldwide 1. Most patients are diagnosed within advanced stage of disease, likely due to the paucity of signs and/or symptoms of early-stage disease. Furthermore, data suggest that approved screening tests for the high-risk patients are not performed optimally 2, 3. Therefore, an earlier diagnosis of HCC patients could result in a substantial improvement in mortality, thereby increasing the number of patients potentially eligible for curative interventions 4, 5.

While more than half of HCC patients are diagnosed during active surveillance, the other newly diagnosed patients are identified when they present with clinical features 6, 7. The presenting clinical features of newly diagnosed HCC patients are highly variable can depend on disease burden and functional status of the liver. Some patients can be completely asymptomatic, or may present with non-specific signs or symptoms, while other patients show features of liver decompensation. Other presenting clinical features may involve signs or symptoms related to mechanical compression, distant metastasis, and/or paraneoplastic syndromes 8.

Previous studies demonstrate that approximately 20% of HCC patients may display diverse clinical and biochemical abnormalities that are not related to disease burden, invasiveness, or distant metastasis. These features can either precede the diagnosis of HCC or present concurrently with HCC. Such paraneoplastic syndromes can appear as the only presenting sign or symptom. It has also been suggested that these manifestations occur secondary to secretion by neoplastic cells of various active molecules, which can exert physical and/or biochemical changes in affected patients. The other proposed hypothesis emphasizes an immune mediated antigen-antibody interaction between neoplastic and normal cells where the newly developed antibodies may cross-react with normal tissues causing damage 9, 10. Amongst various paraneoplastic phenomena, hypoglycemia, hypercalcemia, hypercholesterolemia, erythrocytosis and thrombocytosis are the more frequent abnormalities observed in HCC 11.

Paraneoplastic cutaneous manifestations occur less commonly in HCC patients. In these syndromes, patients present with skin abnormalities secondary to the systemic effect of neoplastic cells, which are not related to the underlying liver disease, distant metastasis, or treatment side effects. Previous studies emphasized that paraneoplastic cutaneous manifestations of HCC are more commonly detected in males and in cirrhotic patients 7, 12. Other studies indicated associations with particular cutaneous abnormalities and some underlying liver diseases 7. However, there is a paucity of published data regarding the clinical significance of paraneoplastic cutaneous manifestations in HCC 13.

In our study, we performed a systematic review and meta-analysis which aimed to explore the association between paraneoplastic cutaneous manifestations in HCC patients and clinical indicators, including outcomes such as survival, response to skin-directed therapy and response to cancer-directed therapy. We also attempted to evaluate the association of paraneoplastic cutaneous syndrome with disease onset, course, underlying liver disease, disease burden and association with distant metastasis.

## Materials and Methods

This study was performed in adherence with the Preferred Reporting Items for Systematic review and Meta-Analyses (PRISMA) guidelines. A systematic search was performed in December 2022 using MEDLINE (host: PubMed and Scopus) and supplemented by a search of Google Scholar. Inclusion criteria included case reports and or case series, involving patients with HCC, who had paraneoplastic cutaneous manifestations at any stage of their disease course. Articles that featured HCC patients with cutaneous abnormalities secondary to other causes, such as direct invasion, distant metastasis or treatment-related side effects were excluded. Review articles and case reports that did not report relevant clinical indicators, such as disease course and response to treatment were also excluded. Each article was screened by two independent reviewers (A.T and D.J). Discrepancy was addressed initially by consensus or if consensus could not be reached with discussion with a third reviewer (L.A.).

Data extraction was performed separately by two reviewers. Extracted data included: name of the first author, year of publication, number of included cases in each study, and country of origin. We additionally reported type, onset, and response to treatment for each reported paraneoplastic cutaneous abnormality. The type of reported paraneoplastic cutaneous feature was extracted from each report as was whether the diagnosis was made based on the result of skin biopsy or by clinical evaluation. Extracted data also included any underlying liver disease, age, sex, and prior alcohol consumption. Disease features such as tumor size, tumor location, disease stage, presence of vascular invasion and/or metastatic disease, alpha fetoprotein (AFP) level, treatment course of HCC, and the temporality between HCC diagnosis and paraneoplastic cutaneous manifestations were also collected. Outcomes of interests included survival (defined by the status of an included patient at the time of reporting rather than at a particular timepoint in follow-up), response to cancer-directed therapy, defined as the observed response to treatment of HCC alone or when the treatment was applied at the same time with skin treatment. Response to skin-directed therapy, defined as observed response to skin treatment alone or when the skin treatment was separated in time from HCC treatment as per investigator assessment. The quality assessment of the included studies was performed using the Joanna Briggs Institute (JBI) critical appraisal framework.

### Data synthesis and statistical analysis

Outcome of interests, including survival, response to cancer-directed and/or skin directed therapy were collected for each included individual. Categorical variables were presented either as counts or percentages, while continuous quantitative variables were displayed using means and range or standard deviation. Data analysis was performed using the Statistical Package for the Social Science (SPSS®) version 25 software (IBM, Armonk NY). The odds ratio (OR) of dichotomous outcomes was calculated using logistic regression. The following variables were explored: age, sex, type and onset of cutaneous manifestation, underlying liver disease, type of skin-directed therapy, stage of HCC, presence of disease recurrence, vascular invasion and distant metastasis, type of cancer-directed therapy, and alfa fetoprotein level. The p-value was calculated using chi-square, for categorical variables, and T-test, for continuous one. Statistical significance was defined as p-value < 0.05. In light of the expected small sample size, multivariable modeling was not performed, and no corrections were made for multiple significance testing. However, quantitative significance as defined by Burnand et al. was explored in addition to statistical significance 14.

### Images of the Paraneoplastic cutaneous manifestations

Images for the most common paraneoplastic cutaneous manifestation of hepatocellular carcinoma were provided in Supplementary Material 1.

## Results

**Figure 1:**
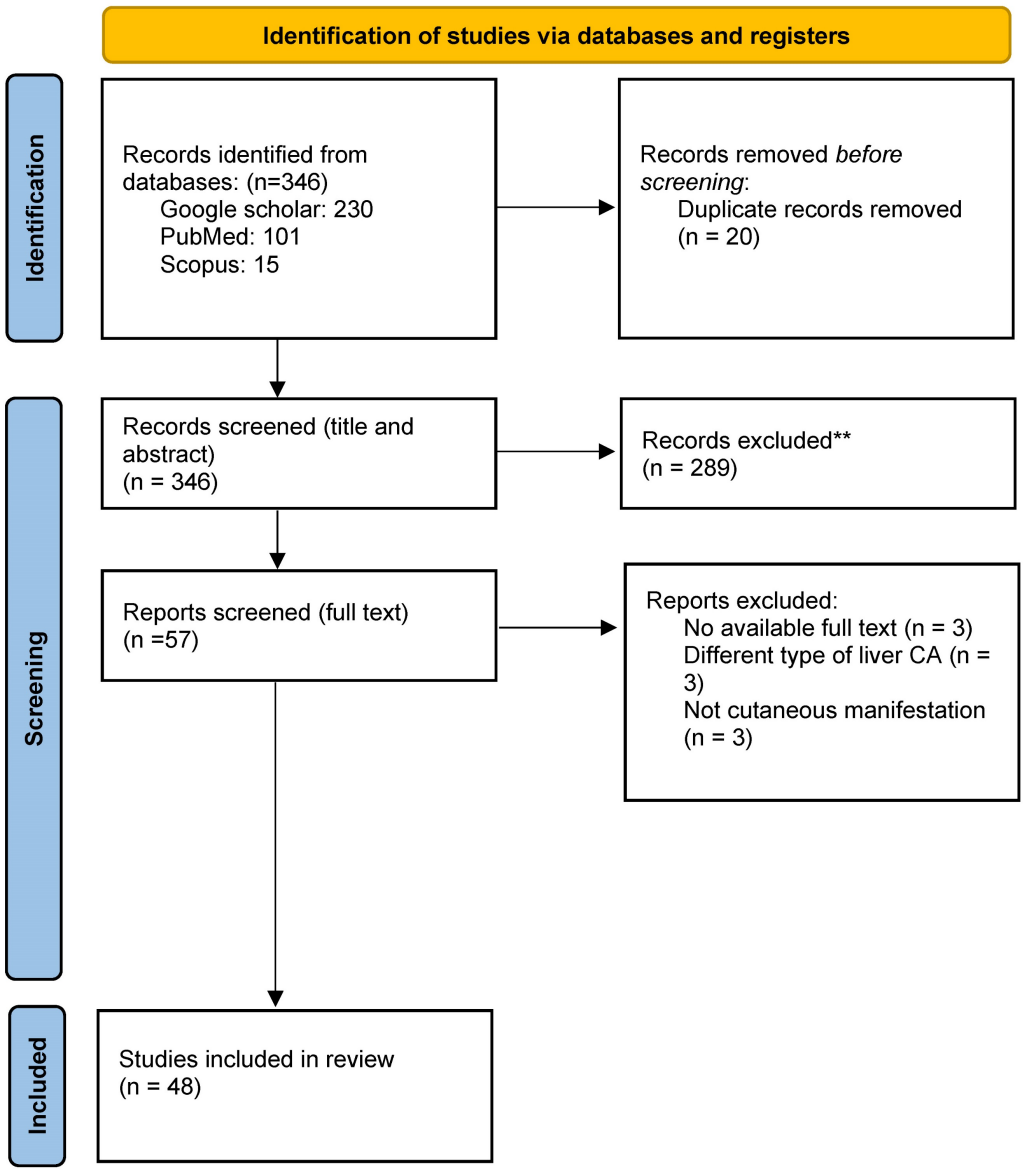
Study selection schema.

A total of 48 studies, comprising 60 patients with HCC were analyzed (see figure 1 for study selection schema) 15-62. Supplementary Table 1 displays the detailed characteristics of the included studies. The median duration of follow up for all included case reports was around 16.4 months (range 2-62 months), while the median age of included patients was 62 years (range 18-90 years). Included studies comprised 50 case reports and one case series. Three-quarters of included patients (n: 45) were males. The most frequent reported skin abnormalities were dermatomyositis (n: 14), pityriasis rotunda (n: 10), and porphyria (n: 7). The majority of patient who presented with dermatomyositis had underlying viral hepatitis, while all reported porphyria and acanthosis cases were associated with metabolic causes of HCC, such as NAFLD (see Table 1). Most skin changes were detected early during the disease course, either shortly before or just after the diagnosis of HCC, while only one sixth of included patients had their paraneoplastic cutaneous manifestations discovered at a late stage or at disease relapse. More than half of the included patients had their skin manifestations as the initial presenting symptom. Only 10 patients (17%) had resectable HCC. Approximately half of the included patients received skin-directed treatment with local or systemic corticosteroids, but only a few required other immunosuppressive therapies. Most skin abnormalities responded to definitive cancer-directed therapy while one out of three patients did not show any response to cancer treatment. Chronic infections with hepatitis B (HBV) and Hepatitis C viruses (HCV) were the most frequently reported underlying liver disease. Reports of paraneoplastic skin changes were more common in patients with metastatic disease compared to those with an early-stage disease. In our study, one out of four patients underwent a definitive locoregional intervention, while one out of three received systemic therapy for an advanced stage HCC. Finally, most reported cases were found in Asia and Europe (see Table 2).

Of note, data regarding outcomes of interests, disease and patient characteristics were not reported explicitly in some of the included case reports. For example, survival data were missing in 18 (30%) patients, while response to cancer-directed and skin-directed therapy were missing in 29 (48%) and 25 (42%) patients respectively. The type of paraneoplastic cutaneous manifestations was not specified clearly in 9 (15%) patients, and the underlying liver disease was not mentioned in 6 (10%) patients. Cancer stage and AFP levels were missing in 7 (12%) and 5 (8%) patients, respectively.

### Death

The probability of death was lower among men as compared to women, a quantitatively significant association which approached, but did not meet statistical significance (OR 0.37, 95% CI: 0.11 to 1.2; p = 0.051). Amongst all reported skin abnormalities, pityriasis rotunda was associated with both quantitative and statistically significantly lower risk of death (OR: 0.05, 95% CI: 0.003 to 0.89; p = 0.04), while patients who presented with dermatomyositis had a quantitatively and statistically significant higher risk of death compared to other paraneoplastic skin manifestations, (OR: 3.37, 95% CI: 1.01-12.1; p = 0.03). As expected, patients who presented with paraneoplastic skin abnormalities concurrently with an early-stage disease had lower odds of death compared to patients who presented with later disease stages (OR: 0.15, 95% CI: 0.028 to 0.76; p = 0.01). The use of local or systemic corticosteroids was associated with better improved survival in patients who had cutaneous abnormalities in which corticosteroids are indicated as a therapy (OR: 0.24, 95% CI: 0.06 to 0.92; p = 0.03). The risk of death was not affected by the underlying cause of HCC. Patients with pre-existing viral hepatitis had similar odds for death, compared to non-viral conditions (OR: 1.1, 95% CI: 0.4 to 3.1; p = 0.43). The onset of skin manifestations appeared to have an impact on the risk of death. Patients who had paraneoplastic cutaneous manifestations prior to HCC diagnosis were quantitatively and statistically more likely to be alive at the time of reporting compared to others (OR: 0.30, 95% CI 0.09 to 0.99; p = 0.02).

### Response of paraneoplastic skin manifestation to skin-directed therapy

Only twelve patients (20%) showed complete response of cutaneous abnormalities to skin-directed therapy, while the same number of patients were observed to have a partial response. With regards to specific paraneoplastic cutaneous manifestations of HCC, no patients with prurigo were observed to respond to skin-directed therapy. Compared to all other skin conditions, prurigo was associated with quantitatively and statistically lower odds of response (OR: 0.04, 95% CI: 0.002 to 0.9, p = 0.04). The odds of improvement of cutaneous lesions, in response to skin-directed therapy, were not affected by age or sex of included patients (OR: 0.99, 95% CI: 0.93 to 1.05, p = 0.51 and 0.65, 95% CI: 0.1 to 4.18, p = 0.69, respectively). There was a quantitatively significant, but statistically non-significant association between use of corticosteroids and improvement in paraneoplastic skin (OR: 0.21 95% CI: 0.04 to 1.11, p = 0.07). The response to skin directed therapy was not quantitatively or statistically different among patients with viral and non-viral underlying liver diseases (OR: 1.87, 95% CI: 0.39 to 9.01, p = 0.43).

### Response of paraneoplastic cutaneous manifestations to cancer-directed therapy

About 20 out of 34 (59%) patients showed an improvement in their cutaneous abnormalities, following cancer-directed therapy. There was a quantitatively significant but statistically non-significant association between the type of cancer-directed therapy and the likelihood of improvement of cutaneous lesions. For example, 7 out of 9 patients who had undergone curative surgical intervention observed improvement in skin lesions (OR 0.32, 95% CI: 0.06 to 1.8, p = 0.19). Conversely, only 2 out of 8 patients, treated with trans arterial chemoembolization (TACE), showed improvement in skin lesions (OR: 3.4, 95% CI 0.6 to 19, p = 0.17). None of the 5 patients who receive chemotherapy showed any resolution in their skin lesions (OR: 12.3, 95% CI 0.63 to 240, p = 0.09). Only 2 out of 7 patients who received multiple therapeutic modalities observed any alleviation in skin lesions OR: 2.8, 95% CI 0.48 to 16.5 p = 0.25.

Again, age and sex did not affect response of skin lesions to cancer directed therapy. Onset and type of paraneoplastic cutaneous skin manifestations had no impact on response to cancer-directed therapy.

## Discussion

Hepatocellular carcinoma is the most prevalent primary liver cancer, representing 90% of all histological subtypes. Worldwide, it is also one of the most common malignancies in both men and women. Consequently, HCC remains a major cause of cancer-related mortality, exceeded only by lung and gastric cancers 63, 64. Paraneoplastic syndromes have been described with HCC, and most features were found to be associated with worse survival, likely as a consequence of an association with a higher burden of disease 65, 66. However, it has also been suggested that specific paraneoplastic features have a predilection to occur early during the disease course while others are detected usually at later stages of the disease 67. HCC is associated with distinctive skin abnormalities, that are not related to distant metastasis or drug toxicity, reflecting a paraneoplastic process. Paraneoplastic cutaneous manifestations represent a wide range of skin abnormalities detected in patients with HCC, which could have an impact on outcomes. However, the clinical significance of paraneoplastic cutaneous manifestations in HCC is not clear. This is likely due to the low prevalence, compared to the other paraneoplastic phenomena that may accompany HCC 68.

Considering the recent introduction of multiple systemic therapies to the treatment armamentarium of advanced HCC, mortality rates have improved considerably 69. However, most of the recently approved agents are either kinase inhibitors or immune checkpoint inhibitors. These agents are known to exert different forms of skin toxicities 70, 71, making the differentiation between paraneoplastic features and treatment-related side effects of great interest, mainly to improve treatment decisions upon the affected patients. In addition, cancer can spread through blood or lymphatics, causing different forms of metastatic skin lesions, where sometimes, reliance on clinical features alone is not enough to establish the potential cause of skin abnormalities, and skin biopsies are required to differentiate between these varying features 72.

Extrapolating from the published data about the effect of non-cutaneous paraneoplastic syndromes 65-67, identification of most paraneoplastic syndromes may indicate a higher disease burden, therefore, we performed a systematic review focusing on HCC patients who had confirmed paraneoplastic cutaneous manifestations at any point during their diseases course.

Our results confirmed a significant association between the onset of paraneoplastic cutaneous manifestation and the odds of death at the time of case reporting, where patients who presented with cutaneous abnormalities prior to HCC diagnosis had more favorable survival. Though this result may have been confounded by lead-time bias. There was a substantial variation in survival among patients who presented with different forms of paraneoplastic cutaneous manifestations, where certain features had worse outcomes than others. As expected, patients who had advanced HCC and patients who did not receive definitive treatment, for HCC, had worse outcomes. In contrast, the use of certain therapeutic agents such as corticosteroids, particularly in those with cutaneous diseases that are managed typically with steroids, was associated with better survival. However, in this observational study, the impact of confounding by indication cannot be excluded especially as the small sample size did not allow for multivariable analyses. Certain types of paraneoplastic cutaneous manifestations did not show any improvement with skin-directed therapy, while most skin changes responded to cancer-directed therapy. Finally, a significant association was observed between certain cutaneous abnormalities and particular liver diseases.

Our study represents an extensive systematic review that examined the clinical significance of different paraneoplastic cutaneous manifestations in HCC patients 8. Paraneoplastic cutaneous manifestations represent a heterogenous group of skin abnormalities, which make it statistically challenging to examine the clinical significance of different cutaneous lesions. In our study, we divided skin abnormalities into subgroups, studying the relative impact of each type compared to the other skin changes. Furthermore, the association between paraneoplastic cutaneous manifestations and other diseases and patients' characteristics were examined.

This study has some limitations. First, there appeared to be various forms paraneoplastic cutaneous manifestations reported in the included case reports, which result in heterogeneity of the summary data. Furthermore, comparison groups were often small resulting in low power to observe statistical significance. While we attempted to explore quantitative as well as statistical significance to address this low statistical power, residual uncertainty will remain. Second, even though our study includes a relatively large number of patients, missing data regarding patients and disease characteristics and outcome of interest were noted in many of the included case reports. This, too, could have affected certainty of our results. Third, we only analyzed the group of patients who had paraneoplastic cutaneous manifestations and HCC. These methods may increase the risk of selection bias. Additionally, we were unable to compare effects of paraneoplastic cutaneous syndrome in affected patients and other HCC patients with no skin involvement and focused instead on differences in outcome and response to therapy between different skin conditions seen in the setting of HCC. Fourth, we acknowledge substantial heterogeneity with lack of standardization in patients' diagnoses and differences in treatment options. We also note that outcomes of interests were not reported consistently amongst all included case reports. To overcome these limitations, we tried to create unified standards in data synthesis and reporting. However, there may be residual uncertainty relating to this heterogeneity. Finally, as time to event data were not reported routinely, the analysis for survival was performed using the status of patients at the time of reporting. This would have resulted in follow-up bias with studies with longer follow-up observing a larger proportion of deaths. However, considering the unfavorable outcomes of HCC patients in general, a median follow-up duration of 29 months would be considered adequate and should minimize the effect of follow up duration on the analysis.

In summary, paraneoplastic cutaneous manifestations represent a heterogenous group of skin abnormalities with a varying prognostic impact on HCC patients. Multiple factors such as onset and type of skin abnormalities, cancer burden, and type of treatment were shown to be associated with outcomes. The results of our study also provide clinicians with useful data about the response to cancer and/or skin directed therapies, which may help clinicians to make better treatment-related decisions in HCC patients who present with paraneoplastic cutaneous manifestations. Further research is needed to examine the effect of paraneoplastic cutaneous manifestations in relation to other paraneoplastic syndromes, and to evaluate the exact burden of paraneoplastic cutaneous manifestation in affected patients compared to other HCC patients.

## Supplementary Material

Supplementary figures and table.

## Figures and Tables

**Table 1:** Correlation between the underlying cause of HCC and types of paraneoplastic cutaneous manifestations.

	Acanthosis	Pemphigus	Dermatomyositis	Disseminated Superficial Porokeratosis	Lesser Trelat	Papuloerythroderma	Pityriasis Rotunda	Porphyria	Prurigo	Others	P-values
NAFLD	5 (17.2)	2 (6.9)	2 (6.9)	0 (0.0)	1 (3.4)	1 (3.4)	3 (10.3)	7 (24.1)	3 (10.3)	5 (17.2)	0.412
HBV	0 (0.0)	1 (5.3)	7 (36.8)	0 (0.0)	1 (5.3)	0 (0.0)	7 (36.8)	0 (0.0)	0 (0.0)	3 (15.8)	0.003*
HCV	0 (0.0)	0 (0.0)	4 (44.4)	3 (33.3)	0 (0.0)	1 (11.1)	0 (0.0)	0 (0.0)	0 (0.0)	1 (11.1)	0.001*
Alcohol	0 (0.0)	0 (0.0)	1 (50.0)	0 (0.0)	0 (0.0)	0 (0.0)	0 (0.0)	0 (0.0)	0 (0.0)	1 (50.0)	0.021*

NAFLD: Non-alcoholic fatty liver disease, HBV: chronic hepatitis B virus infection, HCV: Chronic hepatitis C virus infection.

**Table 2:** characteristics of included case reports

Variable	Response	Frequency (*n* = 60)	Percentage (%)
Sex	Male	45	75.0
	Female	15	25.0
Country	Asia	25	41.7
	America	5	8.3
	Europe	20	33.3
	Africa	10	16.7
Cutaneous Manifestation	Acanthosis	5	8.5
	Pemphigus	3	5.1
	Dermatomyositis	14	23.7
	Disseminated Superficial Parakeratosis	3	5.1
	Lesser Trelat	2	3.4
	Papuloerythroderma	2	3.4
	Pityriasis Rotunda	10	16.9
	Porphyria	7	11.9
	Prurigo	3	5.1
	Others	10	16.9
Onset	Early	50	83.3
	Late	10	16.7
Treatment	Skin and HCC	15	25.0
	HCC Alone	26	43.3
	Skin Alone	19	31.7
Treatment for Cutaneous Manifestations	Corticosteroids	27	45.0
	Azathioprine	4	6.7
	Anti-Histamine	4	6.7
	Others	12	20.0
Response of the Dermatologic Manifestation	None	13	35.1
	Partial	12	32.4
	Complete	12	32.4
Cause of HCC	None-viral, non-alcoholic	30	50.0
	HBV	19	31.7
	HCV	9	15.0
	Alcohol	2	3.3
Recurrence	Yes	8	20.5
	No	31	79.5
Metastasis	Yes	17	63.0
	No	10	37.0
The Correlation between Treatment of HCC and Dermatologic Manifestations	Skin treatment alone or separated by time from HCC treatment	24	49.0
	HCC treatment alone or in the same time with skin treatment	25	51.0
Treatment used for HCC	Surgery	16	26.7
	Radiotherapy	4	6.7
	Systemic Therapy	19	31.7
	Antivirals	4	6.7
	Embolization	7	11.7
Vascular Invasion	Yes	8	88.9
	No	1	11.1
Manifestation Improvement after HCC Treatment	Yes	18	58.1
	No	13	41.9
Correlation between HCC onset and Dermatologic Manifestation	Prior	29	58.0
	Simultaneous	14	28.0
	After	7	14.0
Presenting Symptoms	Yes	34	69.4
	No	15	30.6
Death	Yes	25	62.5
	No	15	37.5
AFP	>400	22	36.7
	<400	33	55.0
Stage	Resectable	10	18.9
	Locally Advanced	27	50.9
	Metastasis	16	30.2
