# Supplementary material for: Paraneoplastic Cutaneous Manifestations of Hepatocellular Carcinoma. A Systematic Review and Meta-analysis
Source: J Cancer. 2024 Jan 1;15(4):1021–9. doi: 10.7150/jca.88931 (PMC10788718; doi:10.7150/jca.88931)
Supplement: Supplementary file 1 — Supplementary figures and table. [file jcav15p1021s1.pdf]

Supplementary material 1: Images of the most common paraneoplastic cutaneous manifestations of hepatocellular carcinoma (HCC):

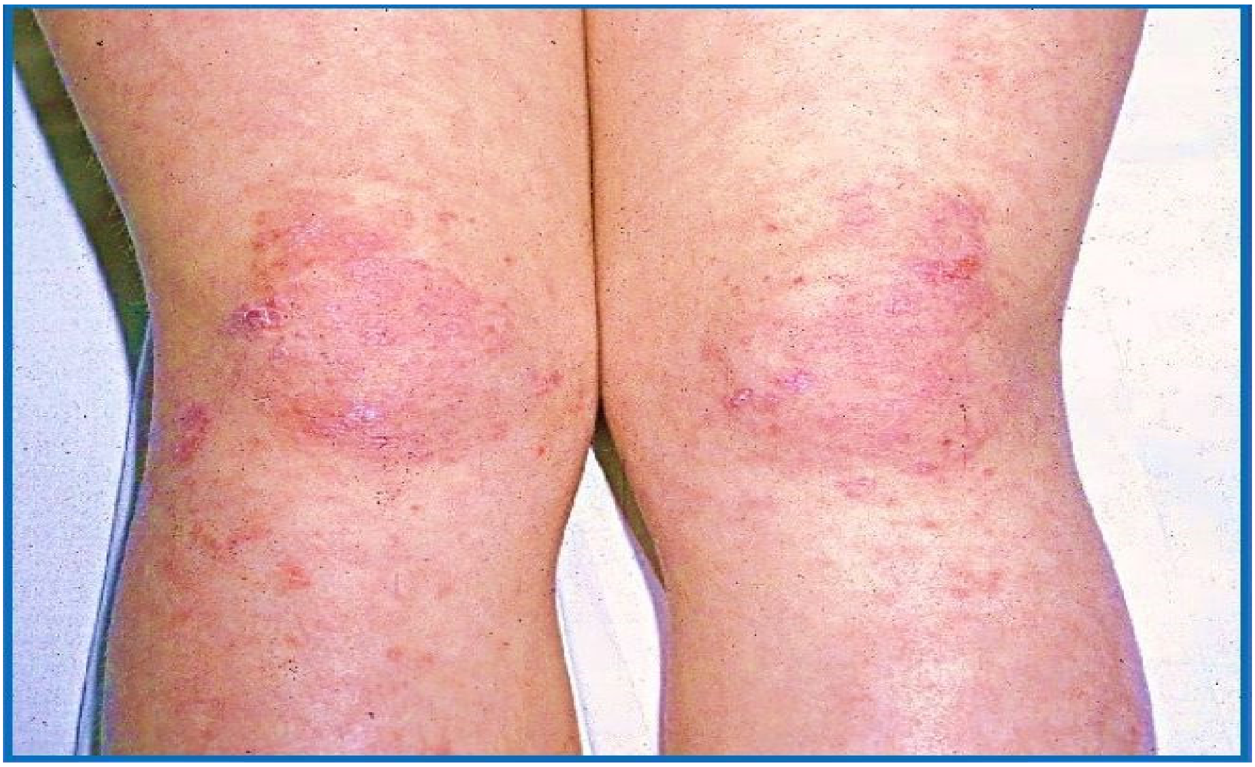

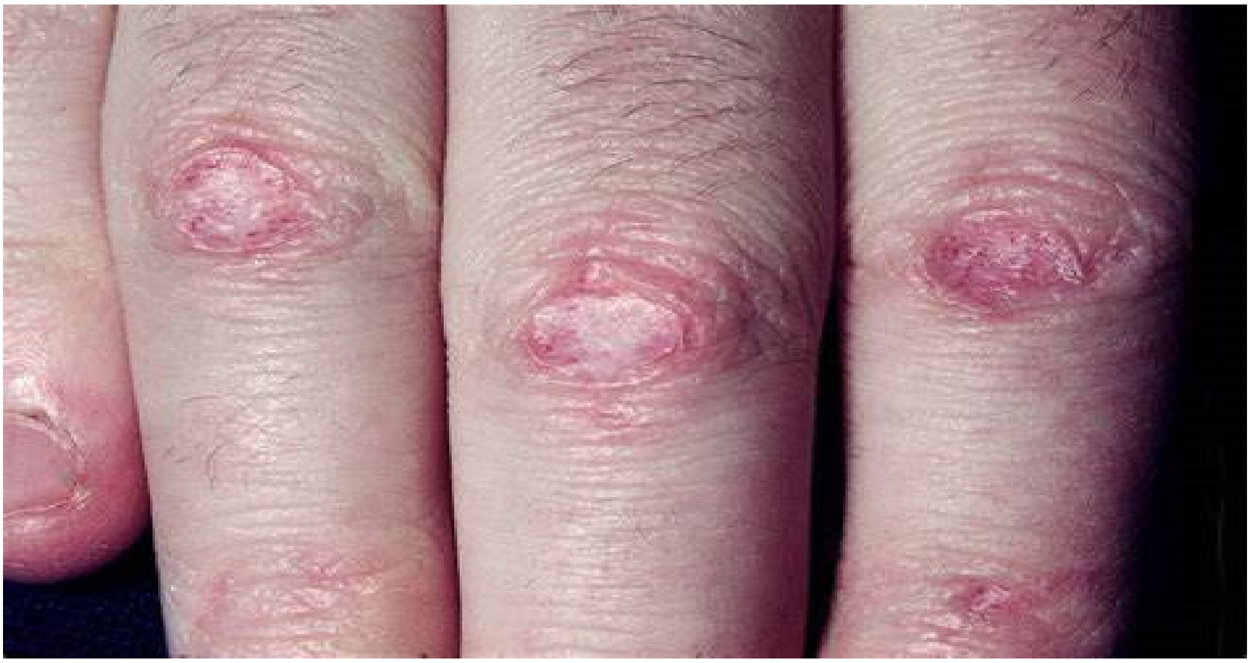

A- Dermatomyositis

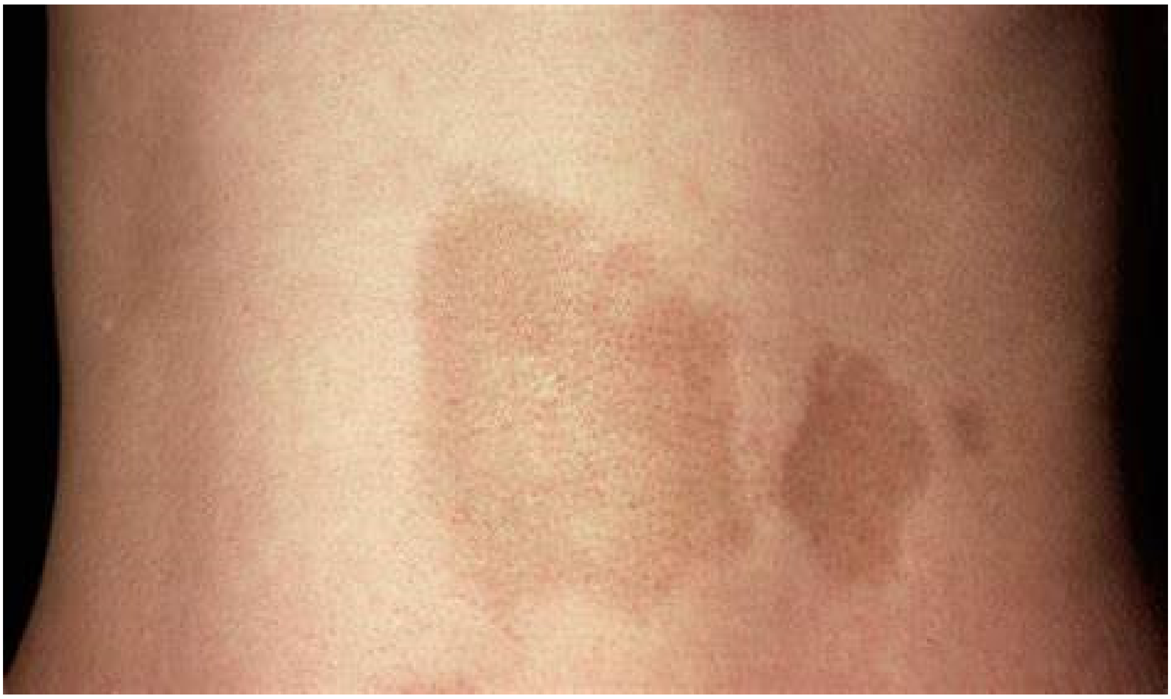

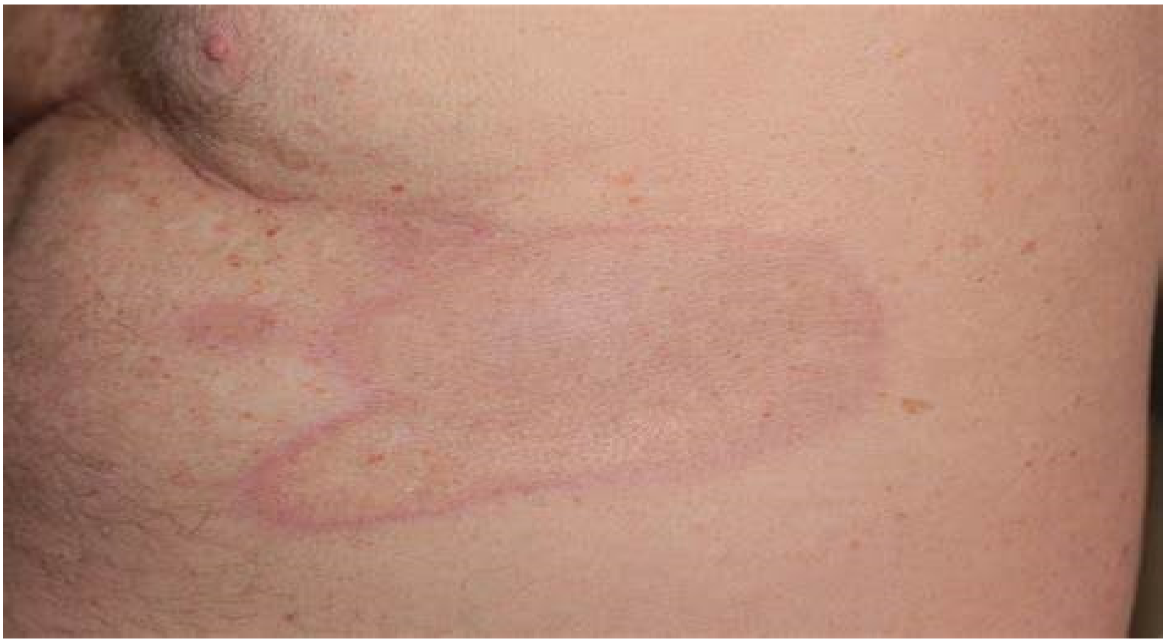

B- Morphea

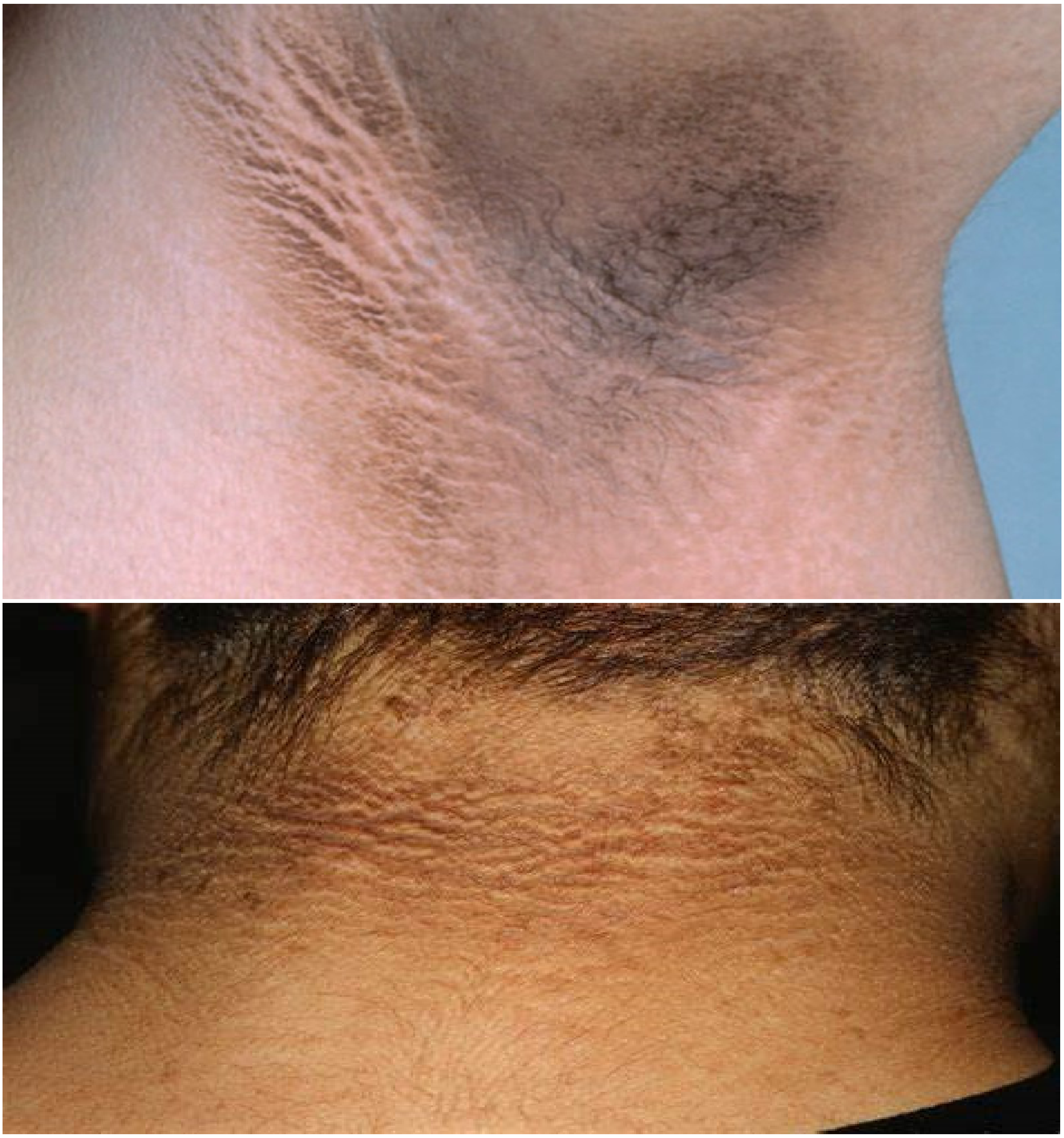

C- Acanthosis Nigricans

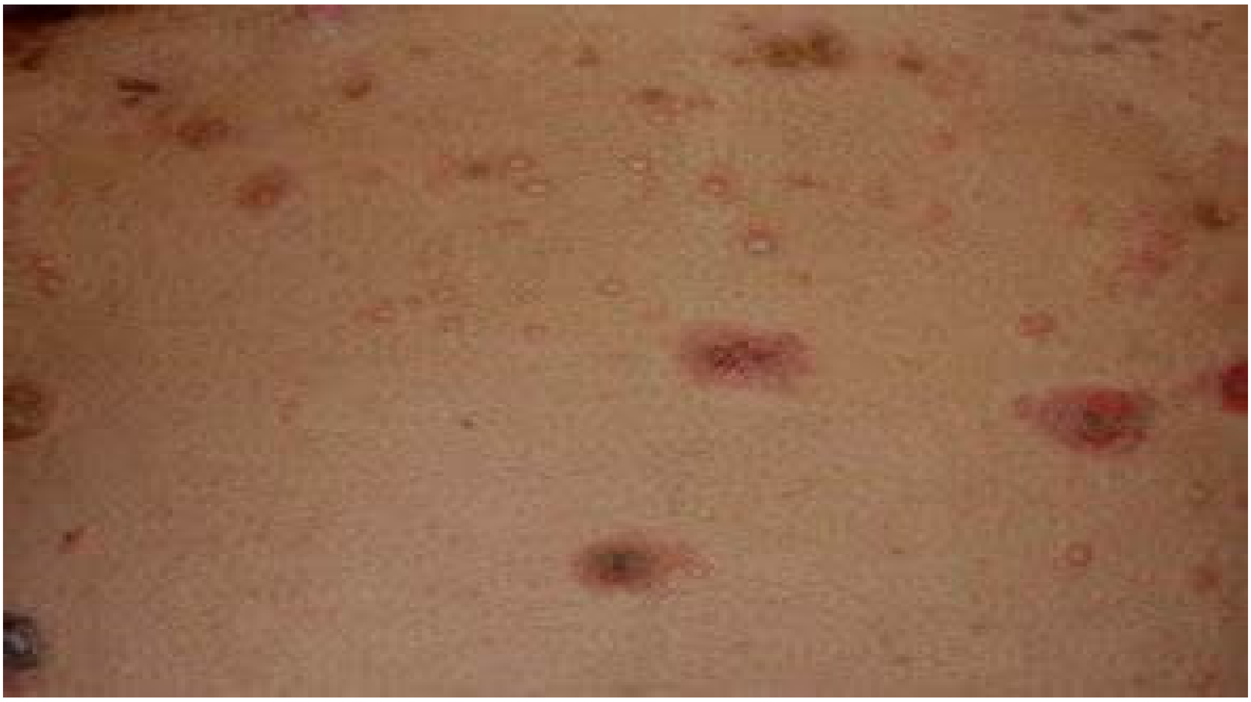

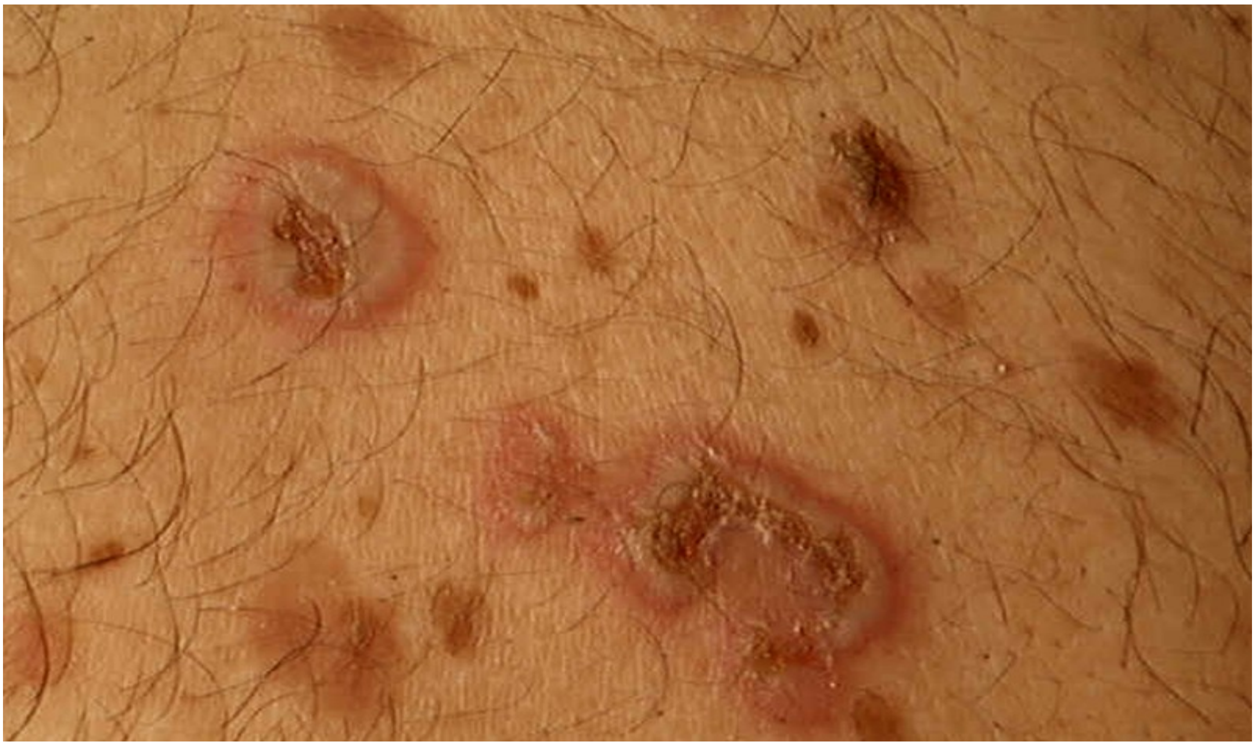

D- Pemphigus Vulgaris

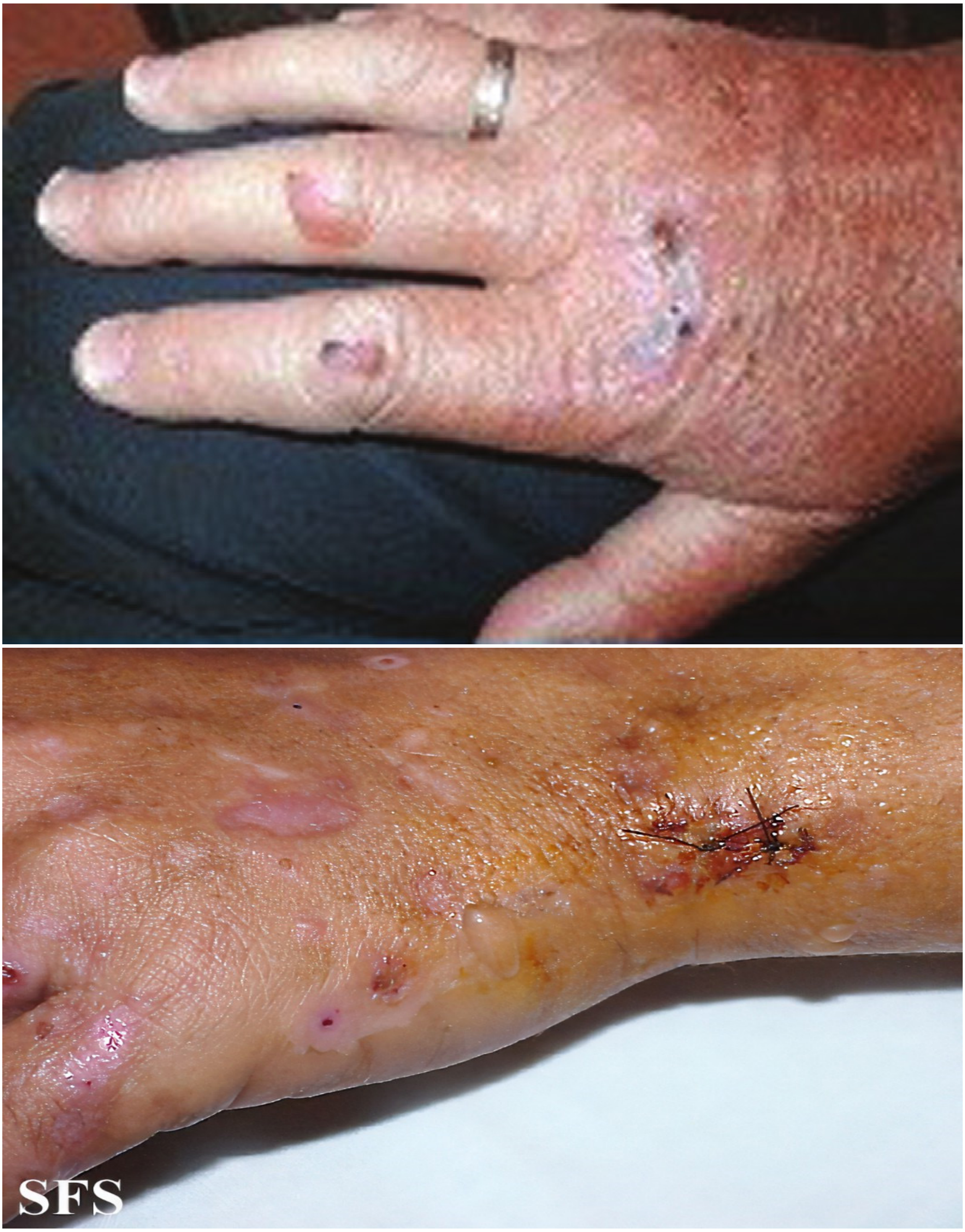

E- Porphyria Cutanea Tarda

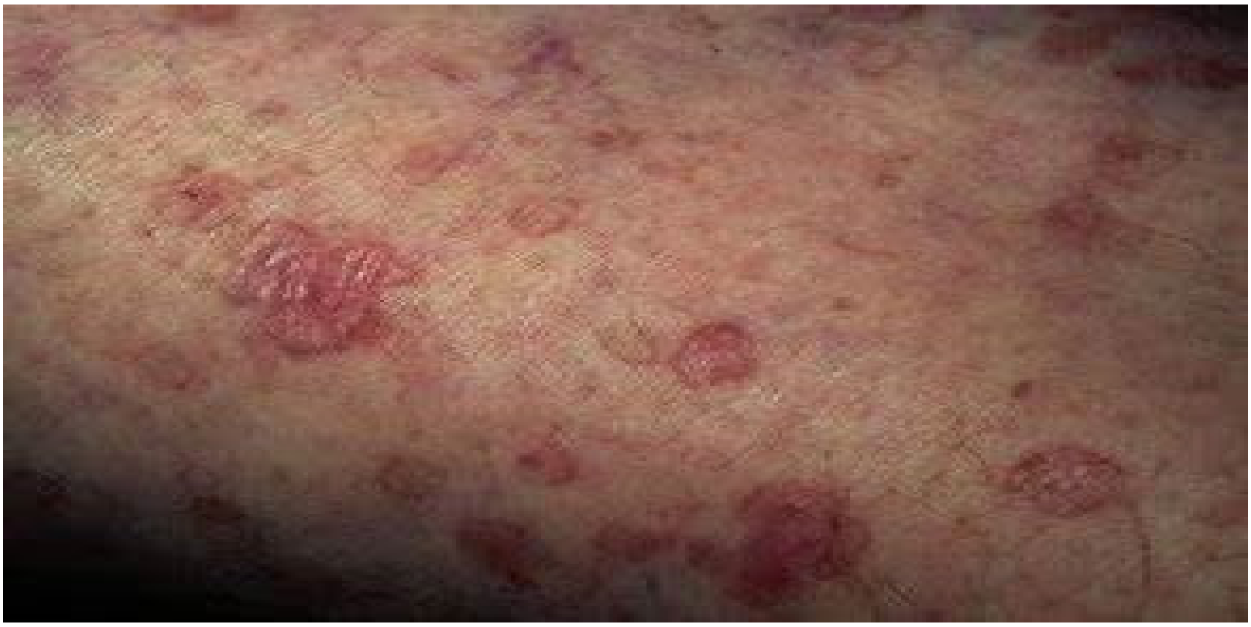

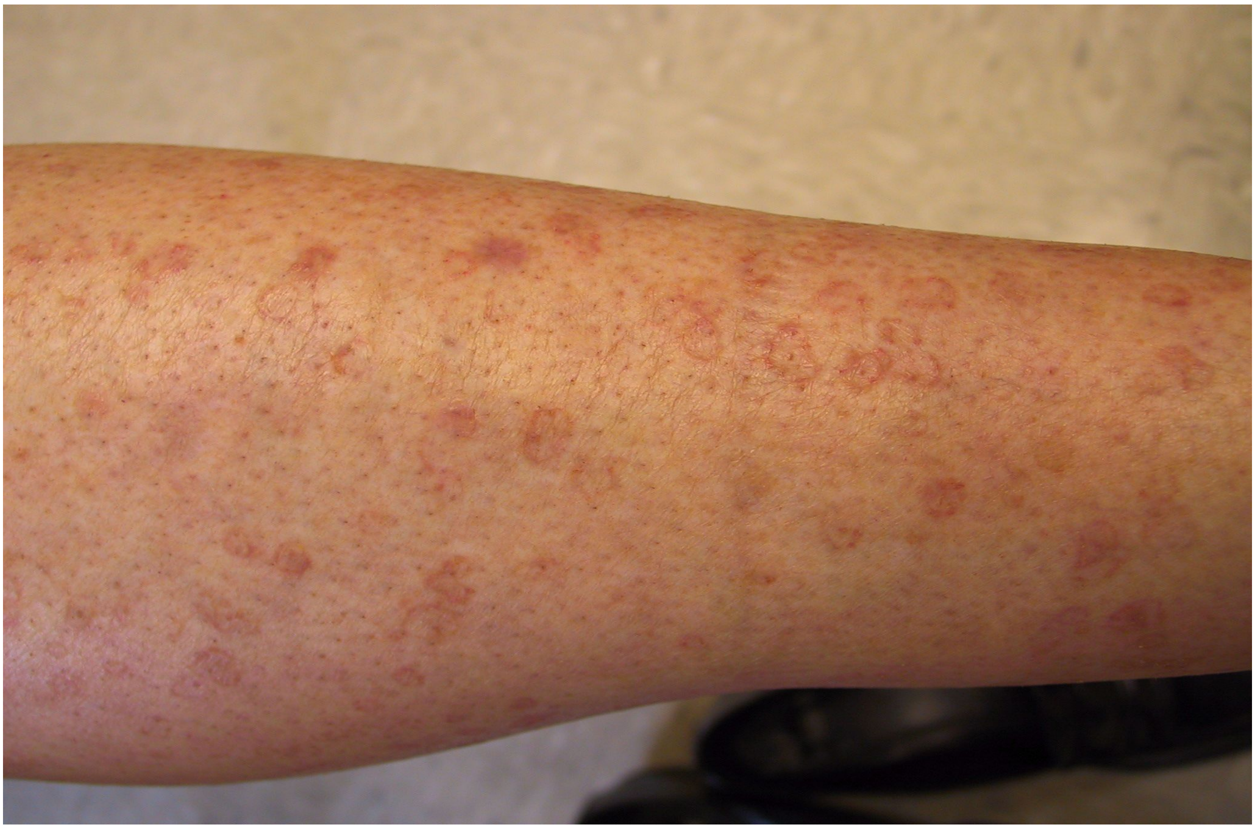

F- Disseminated superficial actinic porokeratosis.

G- Laser Trelat

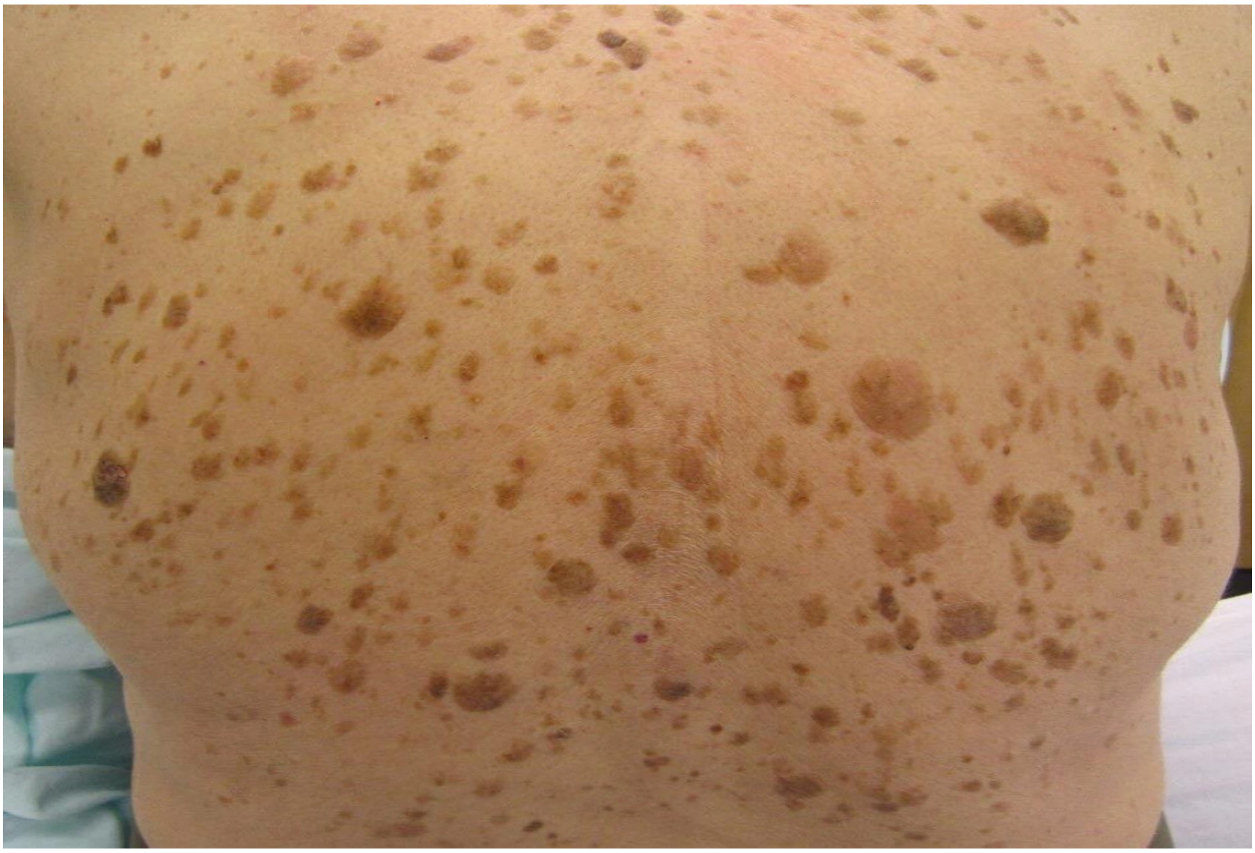

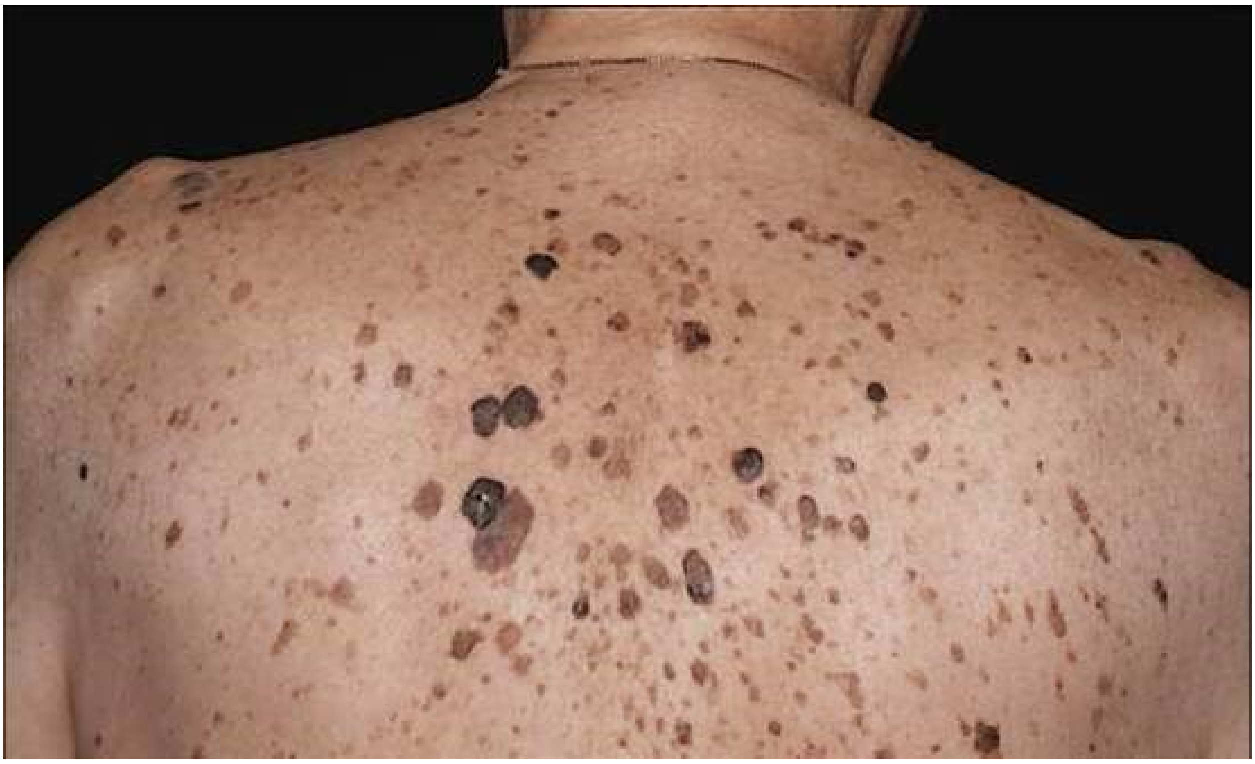

G- Laser Trelat

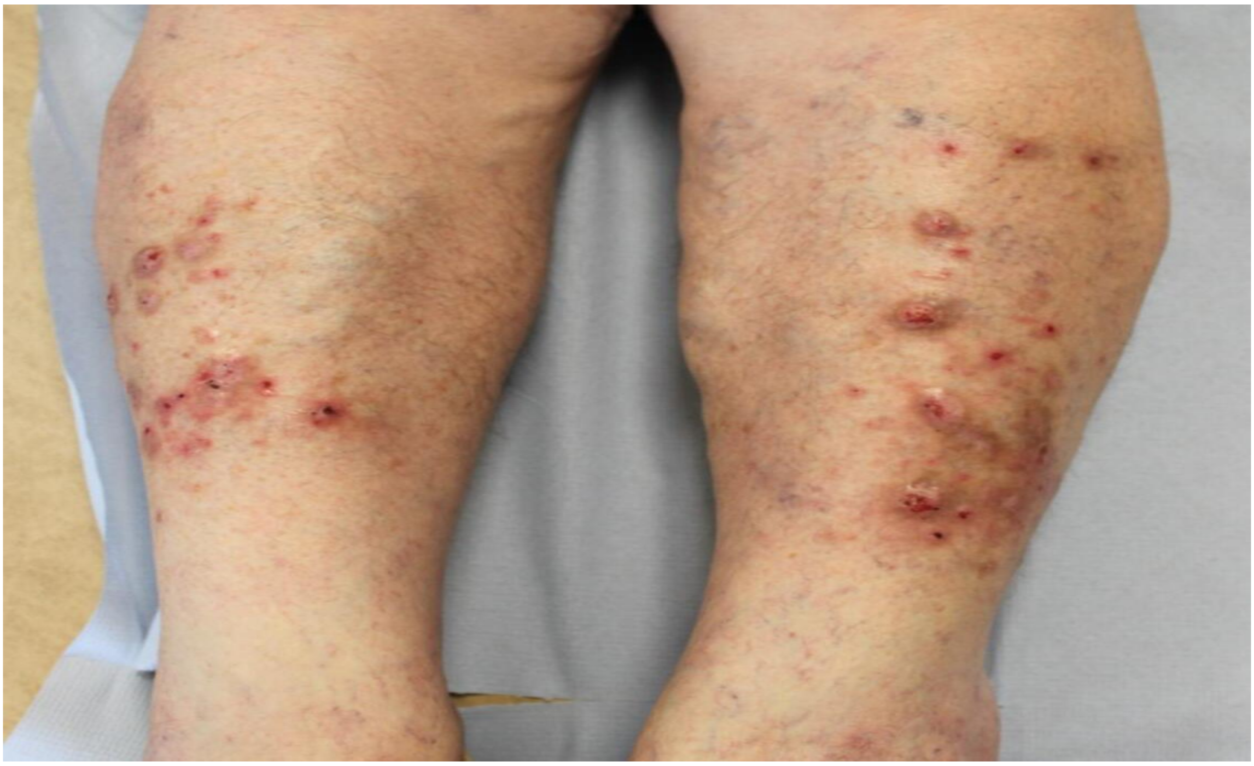

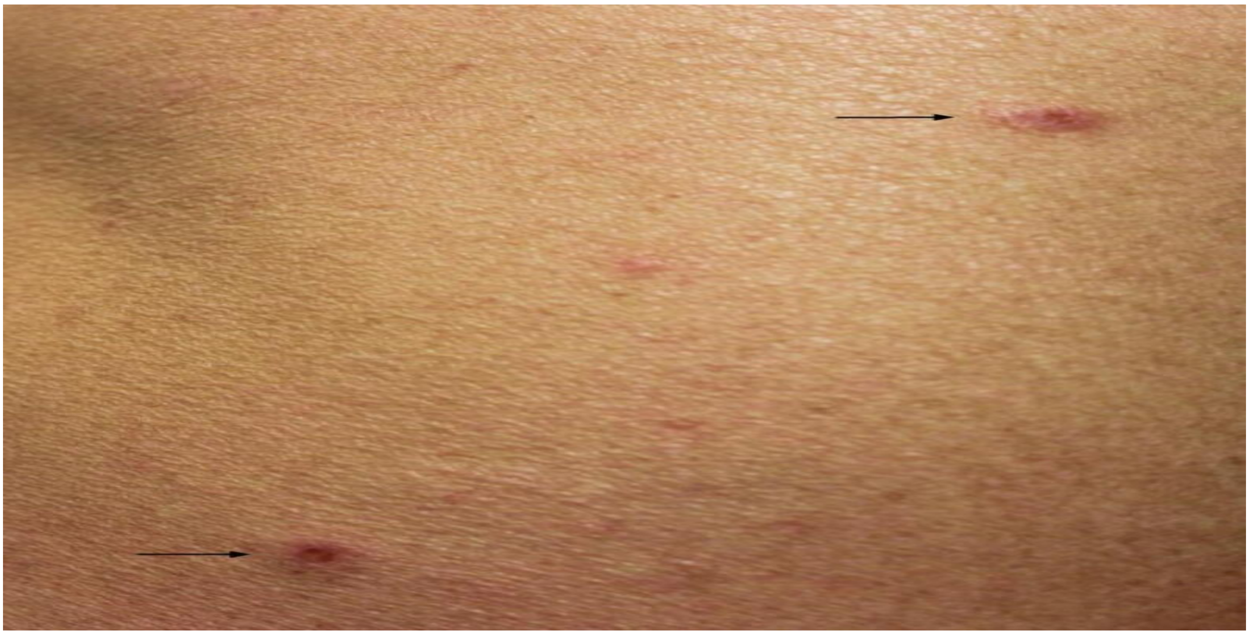

H- Prurigo

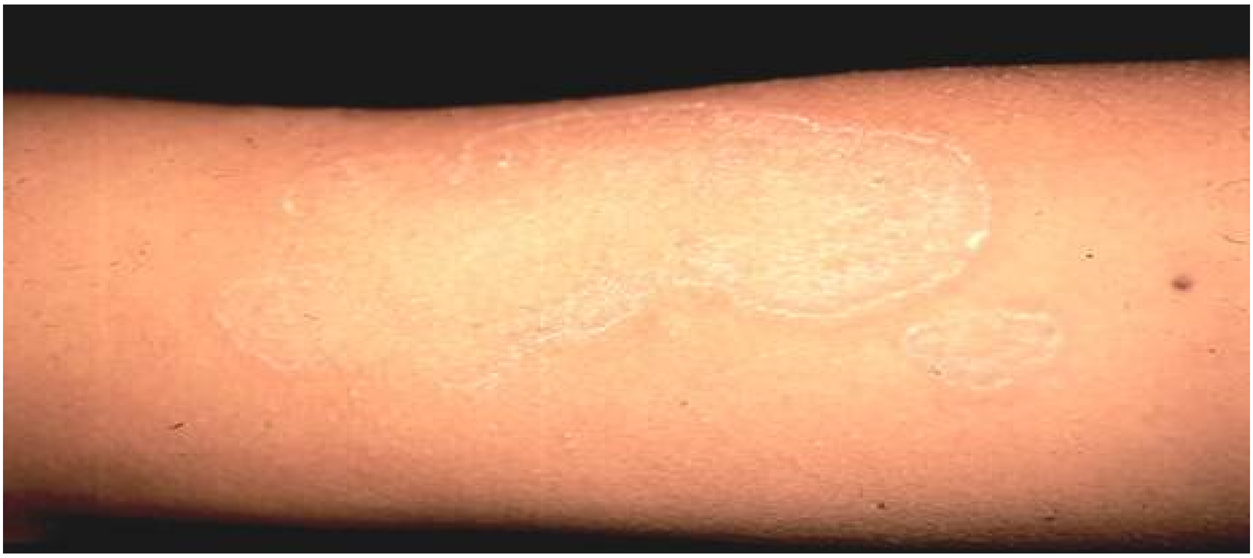

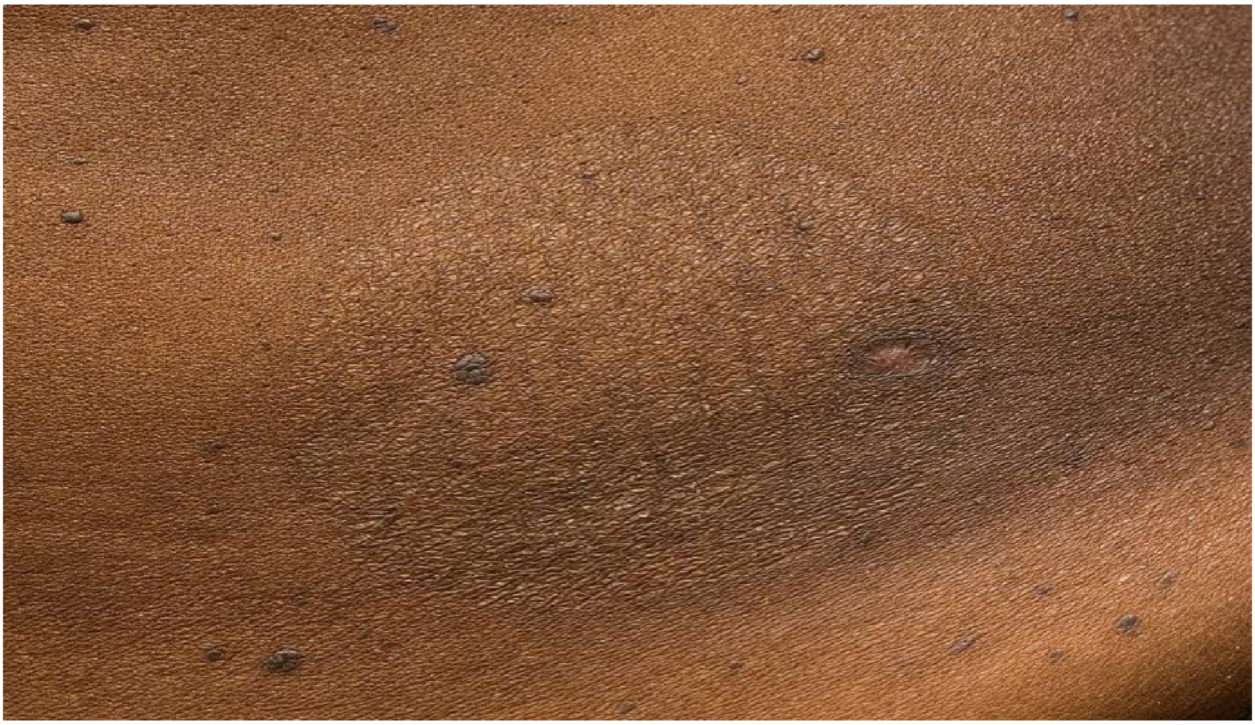

I- Pityriasis Rotunda

Supplementary Table 1 displays the detailed characteristics of the included studies.

1*	2*	3*	4*	5*	6*	7*	8*	9*	10*	11*	12*	13*	14*
Reinaldo Tovo Filho, 2021	Brazil	1	67	Male	Perforating Dermatosis	Early	59 months	Complete response	Alcoholic Cirrhosis	Yes	TACE	Yes	Biopsy
Haruna Goto, 2011	Japan	1	75	Male	Papuloerythroderma of Ofuji and acrokeratosis paraneoplastica (Bazex syndrome)	Early	12 months	No response	N/A	Yes	Surgical resection	N/A	Biopsy
PAPPOVA T, 2017	Slovak Republic	1	68	Male	Morphea	Early	12 months	No treatment	NAFLD	No	N/A	Yes	Biopsy
Juqiang Han, 2018	China	1	62	Male	Dermatomyositis	Early	15 months	Partial response	HBV	Yes	Anti-viral therapy	Yes	Biopsy
Suh Yoon Yang, 2014	Korea	1	55	Male	Dermatomyositis	Early	4 months	Partial response	HBV	No	Anti-viral therapy	Yes	Biopsy
Milagros Cuadros-Torres, 2017	Peru	1	33	Male	Dermatomyositis	Early	7 months	No treatment	HBV	Yes	Surgical resection	N/A	Biopsy
Jen-Wei Chou, 2017	Taiwan	1	55	Male	Dermatomyositis	Early	2 months	Complete response	HBV	Partial	Anti-viral therapy	Yes	Clinical
Sergey Glinkov, 2003	Bulgaria	1	23	Female	Erythema Nodosum	Early	31 months	No response	NAFLD	Yes	Surgical resection	No	N/A
Betsy Ambooken, 2021	India	1	47	Male	Acanthosis Nigricans	Early	12 months	No treatment	NAFLD	N/A	None	N/A	Clinical
Marco Ferronato, 2022	Italy	1	72	Male	Dermatomyositis	Late	60 months	Partial response	HCV	Partial	RFA then TACE	Yes	Biopsy
Naoteru Miyata, 2016	Japan	1	61	Male	Dermatomyositis	Early	1.2 months	Complete response	NAFLD	N/A	None	N/A	Clinical
Soo Ick Cho, 2018	Korea	1	71	Male	Generalized Granuloma Annulare	Early	42 months	No response	HBV	Yes	Sorafenib, TACE, Radiotherapy	Yes	Biopsy
Min-Soo JANG, 2014	Korea	1	54	Male	Pemphigus Vulgaris	Late	36 months	No response	N/A	No	Surgical resection, Radiotherapy, TACE	N/A	Biopsy
Gabriele Hinterhuber, 2003	Austria	1	67	Female	Pemphigus Vulgaris	Late	41 months	Partial response	N/A	No	Surgical resection, TACE, Interferon Alpha	N/A	Biopsy
Hideto YOKOK	Japan	1	58	Male	Pemphigus Vulgaris	Early		Complete response	HBV, HCV	N/A	None	N/A	Biopsy

URA, 2006							38 months						
G Kamińska-Winciorek, 2007	Poland	1	42	Female	Acanthosis Nigricans	Early	22 months	No response	N/A	N/A no tx was done	N/A	N/A	Clinical
S. Sharma, 1997	USA	1	26	Male	Pityriasis Rubra Pilaris	Early	3 months	Complete response	N/A	Yes	TACE	Yes	Biopsy
Hironobu Ihn, 2002	Japan	1	50	Male	Psoriasis	Early	13 months	Partial response	HBV	No	N/A	N/A	Biopsy
Covan Ho, 2001	Australia	1	63	Male	Subacute cutaneous lupus	Late	51 months	Partial response	HBV	No	Surgical resection, cryotherapy, Ethanol injection	N/A	Biopsy
Devireddy Sandeep Reddy, 2021	India	1	18	Male	Acanthosis Nigricans	Early	26 months	No response	NAFLD	No	Surgical resection	N/A	Biopsy
Na Zhang, 2015	USA	1	49	Female	Acanthosis Nigricans	Early	18 months	No response	N/A	No	Chemotherapy	N/A	Clinical
Maria Concetta Fargnoli, 2005	Italy	1	37	Male	Acanthosis Nigricans	Early	27 months	No treatment	N/A	Yes	Surgical resection	No	Clinical
T Muramatsu, 1989	Japan	1	57	Male	Acanthosis Nigricans and Pemphigus Foliaceus	Late	27 months	Complete response	N/A	No	N/A	N/A	Biopsy
Y S Lee , 1996	China	1	69	Male	Perforating Dermatosis	Late	62 months	No response	HBV	Yes	Surgical resection, Chemotherapy, TACE	N/A	Biopsy
K O'Reilly, 1988	UK	1	90	Female	Porphyria Cutanea Tarda	Early	1 month	N/A	N/A	N/A	None	No	Clinical
K Keczkes, 1976	UK	1	60	Female	Porphyria Cutanea Tarda	Early	15 months	Partial response	N/A	No	Chemotherapy	No	Clinical
C I Harrington, 1976	UK	1	72	Male	Porphyria Cutanea Tarda and Laser Trelat	Early	12 months	N/A	N/A	N/A	None	N/A	Biopsy
Monica Corazza, 2003	Italy	1	58	Male	Pancreatic Panniculitis	Early	4 months	Partial response	N/A	No	Chemotherapy	N/A	Biopsy
S.NISHIJIMA 1998	Japan	1	65	Male	Papuloerythroderma	Early	25 months	Partial response	HCV	No	TACE	N/A	Biopsy
Y Horie, 1989	Japan	1	56	Female	Dermatomyositis	Early	15 months	N/A	N/A	N/A	Chemotherapy	No	Clinical
Nobuyuki Toshikuni, 2006	Japan	1	79	Female	Dermatomyositis	Late	13 months	Complete response	HCV	No	TACE	Yes	Biopsy
A Gomez, 1997	Spain	1	73	Male	Dermatomyositis	Early	3 months	Partial response	HCV	No	TACE	N/A	Biopsy
Tsun-I Cheng, 2002	taiwan	1	50	Female	Dermatomyositis	Late	20 months	Complete response	HBV	Yes	TACE then Surgical resection	N/A	Biopsy
Kwong-Ming	taiwan	1	71	Male	Dermatomyositis	Early	1.2 months	Partial response	HCV	N/A	None	Yes	Biopsy

Kee , 2004													
Leonidas Apostolidis, 2009	Germany	1	61	Male	Dermatomyositis	Early	3 months	Complete response	Alcoholic liver disease	Yes	Sorafenib	N/A	Clinical
Seung-Jung Kee, 2009	Korea	1	58	Male	Dermatomyositis	Early	4 months	No response	HBV	N/A	None	Yes	Biopsy
H Tajima, 1991	Japan	1	57	Male	Leser-Trelat	Late	26 months	N/A	HBV	N/A	TACE	Yes	Biopsy
Tiago Mestre, 2014	Portugal	1	68	Male	Disseminated Granuloma Annulare	Early	N/A (died after few months)	partial response	N/A	N/A	TACE	N/A	Biopsy
T Kono, 2000	Japan	3	67	Male	Disseminated Superficial Porokeratosis	Early	50 months	N/A	HCV	Yes	Ethanol injection	N/A	Biopsy
			62	Male	Disseminated Superficial Porokeratosis	Early	6 months	N/A	HCV	Yes	Ethanol Injection	N/A	Biopsy
			58	Female	Disseminated Superficial Porokeratosis	Early	2 months	N/A	HCV	N/A	N/A	N/A	Biopsy
Jiu-Hong Li, 2015	China	1	62	Male	Leser Trelat	Early	42 months	N/A	N/A	No	Surgical resection	N/A	Biopsy
Ahai Luvai, 2015	UK	1	48	Female	Variegate Porphyria	Early	30 months	N/A	N/A	Yes	Surgical resection	No	Clinical
O Dereure, 2000	France	2	71	Male	Prurigo	Early	12 months	No response	N/A	N/A	Chemotherapy	N/A	Biopsy
			74	Male	Prurigo	Late	48 months	No response	N/A	Yes	Surgical resection	No	Biopsy
T Ochiai, 1997	Japan	1	58	Female	Porphyria	Early	24 months	N/A	N/A	N/A	None	No	Biopsy
M. Inuzuka, 2003	Japan	1	51	Male	Acquired Icthyosis and Dermatomyositis	Early	N/A	No response	HCV	N/A	Chemotherapy	N/A	N/A
M J Tidman, 1989	UK	1	70	Female	Variegate porphyria	Early	7 months	N/A	N/A	N/A	N/A	N/A	Clinical
A M DiBisceglie, 1986	South Africa	10	56	Male	Pityriasis Rotunda	Early	24 months	No treatment	HBV	N/A	None	No	Clinical
			63	Male	Pityriasis Rotunda	Early	24 months	No treatment	HBV	N/A	None	Yes	Clinical
			87	Male	Pityriasis Rotunda	Early	24 months	No treatment	HBV	N/A	None	Yes	Clinical
			65	Male	Pityriasis Rotunda	Early	24 months	No treatment	HBV	N/A	None	Yes	Clinical
			67	Male	Pityriasis Rotunda	Early	24 months	No treatment	HBV	N/A	None	Yes	Clinical
			63	Male	Pityriasis Rotunda	Early	24 months	No treatment	N/A	N/A	None	Yes	Clinical
			59	Male	Pityriasis Rotunda	Early		No treatment	N/A	N/A		No	Clinical

							24 months				Chemotherapy		
			50	Male	Pityriasis Rotunda	Early	24 months	No treatment	HBV	N/A	Chemotherapy	Yes	Clinical
			75	Male	Pityriasis Rotunda	Early	24 months	No treatment	HBV	N/A	Chemotherapy	Yes	Clinical
			70	Male	Pityriasis Rotunda	Early	24 months	No treatment	N/A	N/A	Chemotherapy	Yes	Clinical
Carolina Whittle, 2010	Chile	1	69	Male	Porphyria Cutanea Tarda	Early	24 months	Complete response	NAFLD	N/A	Surgical resection, Liver transplant	Yes	Clinical
S Dalle, 2006	France	1	79	Female	Prurigo	Early	15 months	No response	N/A	Yes	Surgical resection	No	Clinical

1: Author and year, 2: Country, 3: Number of cases, 4: Age, 5: Gender, 6: Type of cutaneous manifestation, 7: Onset of cutaneous disease, 8: Follow up duration, 9: Response to skin-directed therapy, 10: Underlying liver disease, 11: Response to cancer-directed therapy, 12: Type of cancer-directed therapy, 13: Liver cirrhosis, 14: Diagnosis of skin conditions.

HCV: Hepatitis C virus, HBV: Hepatitis B virus, NAFLD: Non-Alcoholic Fatty Liver Disease, TACE: Trans-arterial Chemoembolization, RFA: Radiofrequency Ablation.
